# Supplementary material for: Lipid analysis of CO2-rich subsurface aquifers suggests an autotrophy-based deep biosphere with lysolipids enriched in CPR bacteria
Source: ISME J. 2020 Mar 13;14(6):1547–60. doi: 10.1038/s41396-020-0624-4 (PMC7242380; doi:10.1038/s41396-020-0624-4)
Supplement: Supplementary file 1 — Supplementary Material [file 41396_2020_624_MOESM1_ESM.pdf]

SUPPLEMENTARY INFORMATION FOR:

Lipid analysis of CO_2-rich subsurface aquifers suggests an autotrophy-based deep biosphere with lysolipids enriched in CPR bacteria

Running title: Lipidome of a subsurface microbial community

Alexander J. Probst[1,2,#,*], Felix J. Elling[3,4#*], Cindy J. Castelle[1,3], Qingzeng Zhu[3], Marcus Elvert[3], Giovanni Birarda[5,6], Hoi-Ying N. Holman[6], Katherine R. Lane[1], Bethany Ladd[7,§], M. Cathryn Ryan[7], Tanja Woyke[8], Kai-Uwe Hinrichs[3,*], Jillian F. Banfield[1,*]

[1]Department of Earth and Planetary Science, University of California, Berkeley, CA 94720, USA
[2]Insitute for Environmental Microbiology and Biotechnology, Department of Chemistry, University of Duisburg-Essen, Germany
[3]MARUM Center for Marine Environmental Sciences, University of Bremen, Bremen, Germany
[4]Department of Earth and Planetary Sciences, Harvard University, Cambridge, MA 02138, USA
[5]Elettra-Sincrotrone Trieste, Strada Statale 14 - km 163,5, 34149 Basovizza, Trieste, Italy
[6]Molecular Biophysics & Integrated Bioimaging, Lawrence Berkeley National Laboratory, USA
[7]Department of Geoscience, University of Calgary, Calgary, AB, T2N 1N4, Canada
[8]DOE Joint Genome Institute, Walnut Creek, USA

[#]authors contributed equally

*corresponding authors:
alexander.probst@uni-due.de
felix_elling@fas.harvard.edu
khinrichs@uni-bremen.de
jbanfield@berkeley.edu

§present address: Department of Earth, Ocean, and Atmospheric Science, University of British Columbia, Canada, V6T 1Z4

CONTENT: Supplementary Text, Figures, Tables, Data

Extraction and analysis of intact polar lipids.

The filters were stored on dry ice or at -80 °C until extraction. Prior to extraction, filters were cut into 4-cm^2 pieces using combusted (500 °C, 6 h, to remove organic contaminants) and solvent rinsed tools. Lipids were extracted from half of each filter following a modified Bligh and Dyer protocol [1] using combusted glassware and phosphatidylcholine diacylglycerol-$C_{21:0/21:0}$ (Avanti Polar Lipids, Alabaster, AL, USA) as an internal standard. The total lipid extract was dried under a stream of N_2 and stored at -20 °C until measurement. Intact polar lipids (IPLs) were quantified by injecting 10-20% of the total lipid extract dissolved in dichloromethane:methanol (9:1, v:v) into a Dionex Ultimate 3000 ultra-high performance liquid chromatography (UPLC) system connected to a Bruker maXis Ultra-High Resolution quadrupole time-of-flight tandem mass spectrometer equipped with an electrospray ion source operating in positive mode (Bruker Daltonik, Bremen, Germany). Lipids were separated using normal phase UPLC on an Acquity UPLC BEH Amide column (1.7 µm, 2.1 × 150 mm; Waters Corporation, Eschborn, Germany) maintained at 40 °C as described by [2]. The mass spectrometer was set to a resolving power of 27,000 at *m/z* 1,222 and every analysis was mass-calibrated by loop injections of a calibration standard and correction by lock mass, leading to a mass accuracy of better than 1-3 ppm. Ion source and other MS parameters were optimized by infusion of standards into the eluent flow from the LC system using a T-piece.

Lipids were identified by retention time as well as accurate molecular mass and isotope pattern match of proposed sum formulas in full scan mode and MS^2 fragment spectra as described by refs. [1–4]. Structures of detected lipids are shown in Fig. S2. Multistage mass-spectrometric spectra of tentatively identified archaeol containing a hexose-pentose headgroup (1G-1pentose-AR, with G designating a hexose moiety) and of betaine lysolipids are shown in Fig. S3 and Fig. S4. Integration of peaks was performed on extracted ion chromatograms of ±10 mDa width and included the $[M+H]^+$, $[M+NH_4]^+$, and $[M+Na]^+$ ions. Lipid abundances were corrected for headgroup-specific response relative to the internal standard using external calibration curves of commercially available standards. The abundances of diacylglycerol (DAG) lipids with phosphatidylglycerol (PG), phosphatidylethanolamine (PE), and dimethyl PE (PDME) were corrected by the relative responses of commercial DAG-$C_{16:0/16:0}$ standards with the respective headgroup (Avanti Polar Lipids Inc., Alabaster, AL, USA). The abundances of diacylglyceryl-*N,N,N*-trimethylhomoserine (DGTS), diphosphatidylglycerol DAG (DPG), and diglycosyl DAG (DGDG) lipids were corrected by the relative responses of DGTS-$C_{16:0/16:0}$, DPG-$C_{18:1/18:1/18:1/18:1}$, and DGDG-$C_{18:0/18:0}$ (Matreya LLC, Pleasant Gap, PA, USA) standards, respectively. Due to a lack of authentic standards, ornithine and diacylglyceryl-3-*O*-carboxyhydroxymethylcholine (DGCC) lipids were corrected for the response of DGTS-$C_{16:0/16:0}$, respectively. Archaeal lipid abundances were corrected by the relative responses of monoglycosyl archaeol (1G-AR; for 1G-AR and 1G-1pentose-AR) and diglycosyl archaeol (2G-AR; for saturated, monounsaturated, and extended 2G-AR as well as triglycosyl-AR) standards purified from *Archaeoglobus fulgidus* [5]. The lower limit of detection as determined for authentic standards was in the range of 1 to 10 pg for IPLs, depending on compound class and considering a signal-to-noise ratio of greater than 3.

To evaluate potential contamination of filter material with IPLs, blank filters were extracted and analyzed following the same protocol used for samples. No bacterial/eukaryotic or archaeal IPLs were detected in blank filter extracts.

To confirm the applicability of the normal phase UPLC method described above, all samples were additionally screened using the reversed phase method of Wörmer et al. [6]. No additional IPLs were detected using the reversed phase method.

Compound-specific carbon isotopic analysis and mass balance calculations.
Aliquots of the total lipid extracts were separated into two fractions, core lipids and IPLs, using an Agilent 1200 series high-performance liquid chromatography and fraction collection system, as described previously [7]. Ether cleavage was performed on an aliquot of the IPL fraction to release archaeal phytanyl and biphytanyl chains from archaeal lipids, followed by reduction to their hydrocarbon analogues [8]. Another aliquot of IPLs was saponified and derivatized to release and analyze polar lipid-derived fatty acids [9]. Fatty acids were quantified using a gas chromatography-flame ionization detection (GC-FID) system equipped with a Restek Rxi-5ms column (30 m length × 250 mm internal diameter × 0.25 µm film thickness, Restek, Bad Homburg, Germany). Co-injected 2-methyl-octadecanoicacid was used as quantification standard. The GC oven temperature was held at 60 °C for 1 min, increased to 150 °C at a rate of 10 °C min^{-1}, then raised to 310 °C at a rate of 4 °C min^{-1} and held at 310 °C for 40 min. The carrier gas was helium with a constant flow rate of 1.0 ml min^{-1}. The injector temperature was set to 290 °C. Carbon isotopic ratios of fatty acids and archaeal lipid derivatives were determined by GC isotope ratio mass spectrometry (GC-IRMS). Briefly, the samples were injected into a Trace GC Ultra (ThermoFinnigan) equipped with the same column as described above and coupled to a Delta V Plus IRMS via GC IsoLink connected to a ConFlo IV interface (Thermo Fisher Scientific GmbH, Bremen, Germany). The GC's temperature program was the same as used for GC-FID measurements and the oxidation oven of the GC Isolink interface was operated at 1030 °C. The precision of duplicate analyses was ≤0.7‰. All isotopic values are reported in the delta notation as $\delta^{13}C$ relative to the Vienna PeeDee Belemnite (VPDB) standard.

The stable carbon isotope composition of CO_2 was calculated from the stable carbon isotope composition of dissolved inorganic carbon (DIC) using equations from ref. [10] for 15 °C (approximate measured Crystal Geyser borehole water temperature). Stable carbon isotope fractionation from inorganic carbon into lipids was calculated as $\varepsilon_{CO2\text{-}lipid}$ from $\delta^{13}C_{CO2}$-$\delta^{13}C_{lipid}$ or as $\varepsilon_{DIC\text{-}lipid}$ from $\delta^{13}C_{DIC}$-$\delta^{13}C_{lipid}$. The weighted average bacterial $\delta^{13}C_{lipid}$ was calculated from concentrations (from GC-FID) and $\delta^{13}C$ (GC-IRMS) of fatty acids. The maximum expected fractionation for each carbon fixation pathway (Calvin-Benson-Bassham cycle: range 20-30‰ relative to CO_2; reductive tricarboxylic acid cycle: 2-12‰ relative to CO_2; Wood-Ljungdahl cycle: range 30-52‰ relative to DIC;) were adopted from refs. [11–17]. Due to high CO_2 concentrations in the Crystal Geyser subsurface aquifers, $\varepsilon_{CO2\text{-}lipid}$ and $\varepsilon_{DIC\text{-}lipid}$ were assumed to be expressed at the theoretical maximum. The *%CBB cycle vs. rTCA cycle* parameter was calculated from the isotopic mass balance of expected and observed bacterial $\varepsilon_{CO2\text{-}lipid}$. The *%Archaeal autotrophy vs.*

heterotrophy parameter was calculated from the isotopic mass balance of expected and observed archaeal $\varepsilon_{DIC\text{-}lipid}$ and weighted average $\delta^{13}C$ of bacterial lipids (thereby assuming heterotrophic archaeal feed on bacterial biomass, whereas archaeal feeding on archaeal biomass would be isotopically indistinguishable from autotrophy). As lipids and bulk biomass may differ in $\delta^{13}C$ values, a 5‰ uncertainty of ε was assumed for visualization in Fig. 2.

Fourier-transform infrared (FTIR) spectromicroscopy, data processing and analysis.
Recovered cells that passed through a 0.2-µm filter but retained by a 0.1-µm filter were deposited on a double-side-polished silicon slide and dried with a gentle nitrogen gas stream in a biological safety cabinet. All measurements were made on the silicon slide in conjunction with the Hyperion 3000 Infrared-Visible microscope that was coupled to a Vertex70V interferometer (Bruker Optics – Billerica MA) from the Berkeley Synchrotron Infrared Structural BioImaging (BSISB) program. The microscope was equipped with a 128×128 pixels focal plane array detector. Together with a 15× magnification objective, each individual image field (or tile) covers an area of ~300×300 µm. Nine tiles where measured, collecting a total of ~150,000 spectra. Each spectrum represents an average of 1024 scans acquired at the mid-infrared frequency range between 900 and 3,700 cm^{-1} at a spectral resolution of 4 cm^{-1}.
All FTIR spectra were corrected for CO_2 and water vapor with a built-in function "OPUS7.5" (Bruker Optics – Billerica MA), exported in ENVI format to R-Studio, which were analyzed using the following three R packages: Hyperspec [18], Signal [19] and Baseline [20]. In this study, data were first baseline corrected with a "fillpeaks" function of the baseline package. Then, we removed spectra from pixels that did not have cells by applying an intensity filter on the 3200 cm^{-1} signal to filter out all the pixels with intensity lower than 0.02 a.u (abundance units). This yielded a final dataset of 89,000 spectra. A principal component analysis (PCA) was first performed on the 2800-3050 cm^{-1} energy range to focus on differences in lipid spectral features. Loading vectors of this latter analysis were compared with spectra of dry films of reference lipids: 1-oleoyl-2-stearoyl-sn-glycero-3-phosphocholine (C18:1-18:0-PC) versus 1-oleoyl-2-hydroxy-sn-glycero-3-phosphocholine (lyso C18:1-PC). Both C18:1-18:0-PC and lyso C18:1-PC were purchased from Avanti Polar Lipids, Inc. (Alabaster, Alabama, USA). To prepare lipid films, 1.0 mg of C18:1-18:0-PC or lyso C18:1-PC were dissolved in ~200 µl of chloroform, then deposited ~5 µl of the solute droplets onto a double-side-polished silicon slide and dried under a gentle N_2 stream for 1 hour before FTIR measurement. A second PCA was then performed over the whole 900-3700 cm^{-1} spectral range to determine whether the variance of the spectral patterns highlights the main chemical composition of the samples.

Additional FTIR data analysis
Additional principal component analysis on the 900-3700 cm^{-1} spectral range was performed to gather more information on the chemical environment that harbored CPR cells. The results showed that >95% of the variance for both samples can be represented by the first 5 components (Fig. S9).

The sample presents strong signals in the lower wavenumber region, between 1200 and 900 cm^{-1}. Loading vector 1 is characterized by a strong signal at 1125 cm^{-1} characteristic for sulfates, in particular iron and manganese sulfates. Moreover, loading vector 1 presents signals at 1680 and 1540 cm^{-1}, assigned to proteins as amide I and amide II respectively; the signal at 1680 cm^{-1} is quite broad and has a shoulder at 1633 cm^{-1}, due to beta sheet conformation of proteins, and another shoulder at 1725 cm^{-1} assigned to the carbonyl group of lipids. Loading vector 2 shows strong absorption at 1415 cm^{-1} due to presence of calcium carbonate and two sharp signals at 2920 cm^{-1} and 2850 cm^{-1} originating from CH_2 of lipid aliphatic chains. Loading vector 3 exhibits a strong peak at 1125 cm^{-1}, but no other relevant features, whereas loading vector 4 shows two sharp signals from CH_2 of the lipid aliphatic chains at 2920 cm^{-1} and 2850 cm^{-1} and a signal at 2950 cm^{-1} from CH_3, suggesting the presence of a different type of lipid containing more branching, possibly archaeal isoprenoid lipids. In summary, the chemical environment is rich in sulfates, carbonates, and silicates. The cellular material exhibits signals of proteins, folded in beta-sheet structures and a mixture of signals from lysolipids with different levels of branching and unsaturation, belonging to bacterial and archaeal cells.

Analysis of dissolved organic carbon

Water samples were collected for dissolved organic carbon (DOC) in July 2019 during the minor eruption phase of Crystal Geyser. Samples were filtered through 0.1 µm syringe filters, acidified with hydrochloric acid in the field, and transported and stored at 4 °C. The samples were sparged with helium in the lab prior to being loaded onto a model 1088 autosampler for analysis. Quantification and carbon isotopic analysis of DOC was performed following the protocol of Lalonde et al. [21] and using a OI Analytical Aurora 1030W TOC Analyzer interfaced to a Finnigan Mat DeltaPlusXP IRMS. The 2σ analytical precision was 2% (of the ppm value) for quantification and ±0.2‰ for carbon isotopic composition.

Diversity and phylogeny of 3-oxoacyl-[acyl-carrier protein] reductase beta subunit

To investigate the diversity of dehydrogenases involved in fatty acid reduction, we picked the 3-oxoacyl-[acyl-carrier protein] reductase beta subunit as a representative example. 67 currently existing assemblies from Crystal Geyser [22–24] underwent gene prediction using prodigal [25] followed by an HMM search [26] using the respective model from KEGG K11539 [27] with an e-value cutoff of 10^{-10}. Results were annotated against UniRef100 (e-value cutoff 10^{-5}) and only those results were retained, which contained the annotation strings “3-oxoacyl-ACP_reductase”, “3-oxoacyl-_acyl-carrier-protein__reductase”, “Beta-ketoacyl-ACP_reductase”, “Enoyl-_acyl-carrier-protein__reductase__NADH”, “Enoyl-_acyl-carrier-protein__reductase”, “3-oxoacyl-_Acyl-carrier_protein__reductase”, “3-oxoacyl-_acyl-carrier_protein__reductase”, and “3-ketoacyl-ACP_reductase”. The resulting sequences were clustered using CD-Hit [28] at 90% amino acid similarity and aligned using muscle [29]. After manual end-trimming of the alignment a tree was computed using FastTreeMP [30] with default parameters. Visualization of the tree was done using the iTOL software [31].

Table S1. Metagenomic data included in this study.

Sample	Year	Geyser phase	Filter in µm | volume filtered in L	Used for	SRA Accession	Reference
CG01*	2015	recovery	0.1 | 21	metagenomics correlation	SRS2524913	Probst et al., 2018
CG02	2015	recovery	0.1 | 57	metagenomics correlation	SRS2524943	Probst et al., 2018
CG03	2015	recovery	0.1 | 150	metagenomics correlation	SRS2524942	Probst et al., 2018
CG04	2015	recovery	0.1 | 114	metagenomics correlation	SRS2524959	Probst et al., 2018
CG05	2015	recovery	0.1 | 189	metagenomics correlation	SRS2524997	Probst et al., 2018
CG06	2015	recovery	0.1 | 95	metagenomics correlation	SRS2524958	Probst et al., 2018
CG07	2015	recovery	0.1 | 170	metagenomics correlation	SRS2524998	Probst et al., 2018
CG08	2015	transition	0.1 | 151	metagenomics correlation	SRS2524996	Probst et al., 2018
CG09	2015	transition	0.1 | 168	metagenomics correlation	SRS2525178	Probst et al., 2018
CG10	2015	transition	0.1 | 159	metagenomics correlation	SRS2525213	Probst et al., 2018
CG11	2015	minor eruptions	0.1 | 227	metagenomics correlation	SRS2525260	Probst et al., 2018
CG12	2015	minor eruptions	0.1 | 136	metagenomics correlation	SRS2525262	Probst et al., 2018
CG13	2015	minor eruptions	0.1 | 169	metagenomics correlation	SRS2525263	Probst et al., 2018
CG14	2015	minor eruptions	0.1 | 151	metagenomics correlation	SRS2525370	Probst et al., 2018
CG15	2015	minor eruptions	0.1 | 148	metagenomics correlation	SRS2525432	Probst et al., 2018
CG16	2015	minor eruptions	0.1 | 151	metagenomics correlation	SRS2525532	Probst et al., 2018
CG17	2015	minor eruptions	0.1 | 155	metagenomics correlation	SRS2525582	Probst et al., 2018
CG18	2015	minor eruptions	0.1 | 142	metagenomics correlation	SRS2525587	Probst et al., 2018
CG19	2015	transition	0.1 | 114	metagenomics correlation	SRS2525664	Probst et al., 2018
CG20	2015	transition	0.1 | 93	metagenomics correlation	SRS2525665	Probst et al., 2018
CG21	2015	major eruptions	0.1 | 189	metagenomics correlation	SRS2525667	Probst et al., 2018
CG22	2015	major eruptions	0.1 | 151	metagenomics correlation	SRS2525670	Probst et al., 2018
CG23	2015	major eruptions	0.1 | 132	metagenomics correlation	SRS2525668	Probst et al., 2018
CG24	2015	major eruptions	0.1 | 170	metagenomics correlation	SRS2525671	Probst et al., 2018
CG25	2015	transition	0.1 | 170	metagenomics correlation	SRS2525666	Probst et al., 2018
CG26A	2015	recovery	0.2 | 379**	metagenomics correlation	SRS2525669	this study
CG26B	2015	recovery	0.1 (post 0.2)**	metagenomics correlation	SRS2527020	this study
CG10_big_fil_rev_8_21_14_0.10	2014	recovery	0.1 (post 0.2)	metagenomics coupled to IR	SRS2647932	Probst et al., 2018

*sample underwent whole genome amplification for sequencing

** volume of CG26A and CG 26B is identical as these were sequential filters

Table S2. Samples for lipidomic analyses and the respective volume of groundwater which was filtered.

Sample	Vol in L
CG-0.1um-2015-05-25-7:30-teflon-01	114
CG-0.1um-2015-05-25-16:30-teflon-04	303
CG-0.1um-2015-05-26-00:30-teflon-06	299
CG-0.1um-2015-05-26-09:00-teflon-08	322
CG-0.1um-2015-05-26-17:00-teflon-10	333
CG-0.1um-2015-05-27-03:00-teflon-12	388
CG-0.1um-2015-05-27-11:00-teflon-14	358
CG-0.1um-2015-05-27-18:51-teflon-16	360
CG-0.1um-2015-05-28-02:21-teflon-18	314
CG-0.1um-2015-05-28-10:00-teflon-20	265
CG-0.1um-2015-05-28-20:49-teflon-22	360
CG-0.1um-2015-05-29-04:53-teflon-24	341
CG-0.1um-2015-05-29-7:36-teflon-25	114
CG-0.1um-post0.2-2015-05-29-19:30-teflon-01	379

Table S3. Concentration and carbon isotopic composition of dissolved inorganic carbon (DOC) during minor eruptions of Crystal Geyser.

Sample name	Collection date	[DOC] (ppm)	$\delta^{13}C_{DOC}$
CG 0712-1	07/12/2019 14:24	1.6	-19.4
CG 0712-2	07/12/2019 14:27	1.4	-24.0
CG 0712-3	07/12/2019 18:22	1.4	-22.6
CG 0712-4	07/12/2019 18:24	1.2	-21.9
CG 0713-5	08/12/2019 06:44	1.0	-21.2
CG 0713-6	08/12/2019 06:46	1.0	-23.6
CG 0713-7	08/12/2019 09:02	1.1	-22.6
CG 0713-8	08/12/2019 09:05	1.5	-24.0

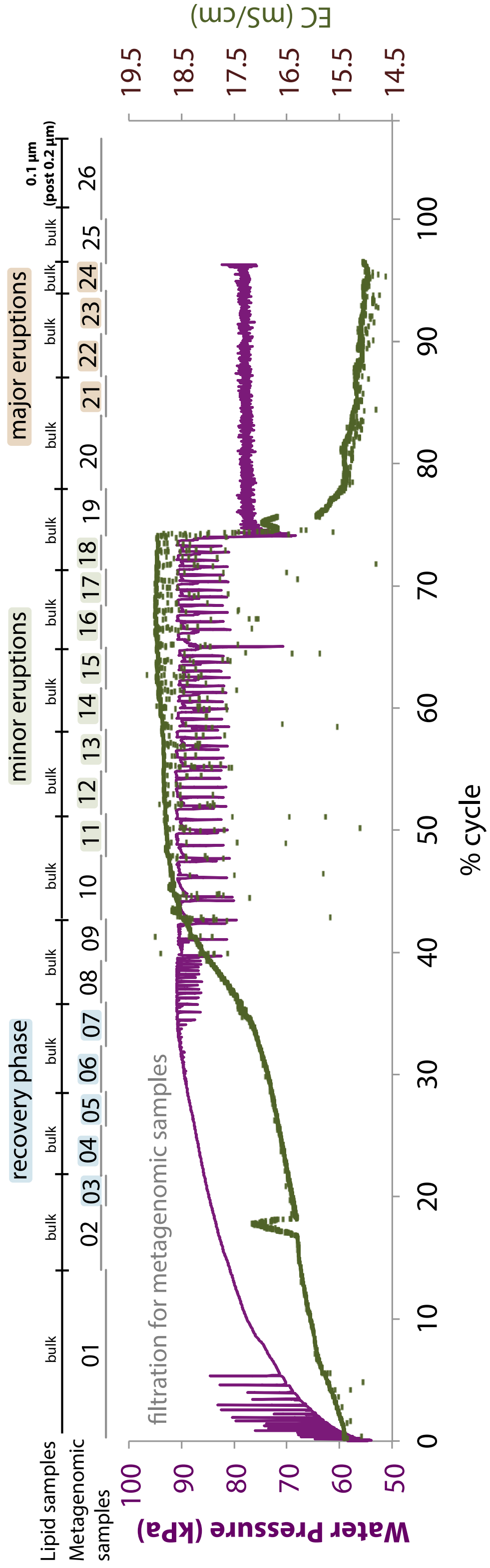

Fig. S1 | Sampling scheme of the near 5-day eruption cycle as previously displayed and explained in [22]. Please note the alignment of the metagenomic samples (displayed as numbers) and the lipidomic samples that were taken in parallel. For most of the samples, two metagenome samples are representative of one lipidomic sample. Bulk = collection of fluids directly onto a 0.1 µm filter. Sample 26 was a sequential filtration first onto a 0.2 µm filter, then onto a 0.1 µm filter.

One additional sample, collected outside of this field campaign was also included for metagenomic and infrared analysis. This particular sample was collected in the middle of the recovery phase, when the abundance of CPR was high and little to no *Sulfurimonas*, which passes through 0.2 µm filter, was present.

Fig. S2 | Structures of bacterial and archaeal membrane lipids identified in Crystal Geyser samples. Intact polar lipids consist of a polar headgroup connected to an apolar core lipid. Note that not all possible combinations were detected in Crystal Geyser. The type of hexoses and pentoses could not be distinguished by the employed analytical techniques and the exact structure of the 1G-1pentose headgroup is unknown (i.e., whether it is the hexose or the pentose that is bound to the glycerol moiety of the core lipid).

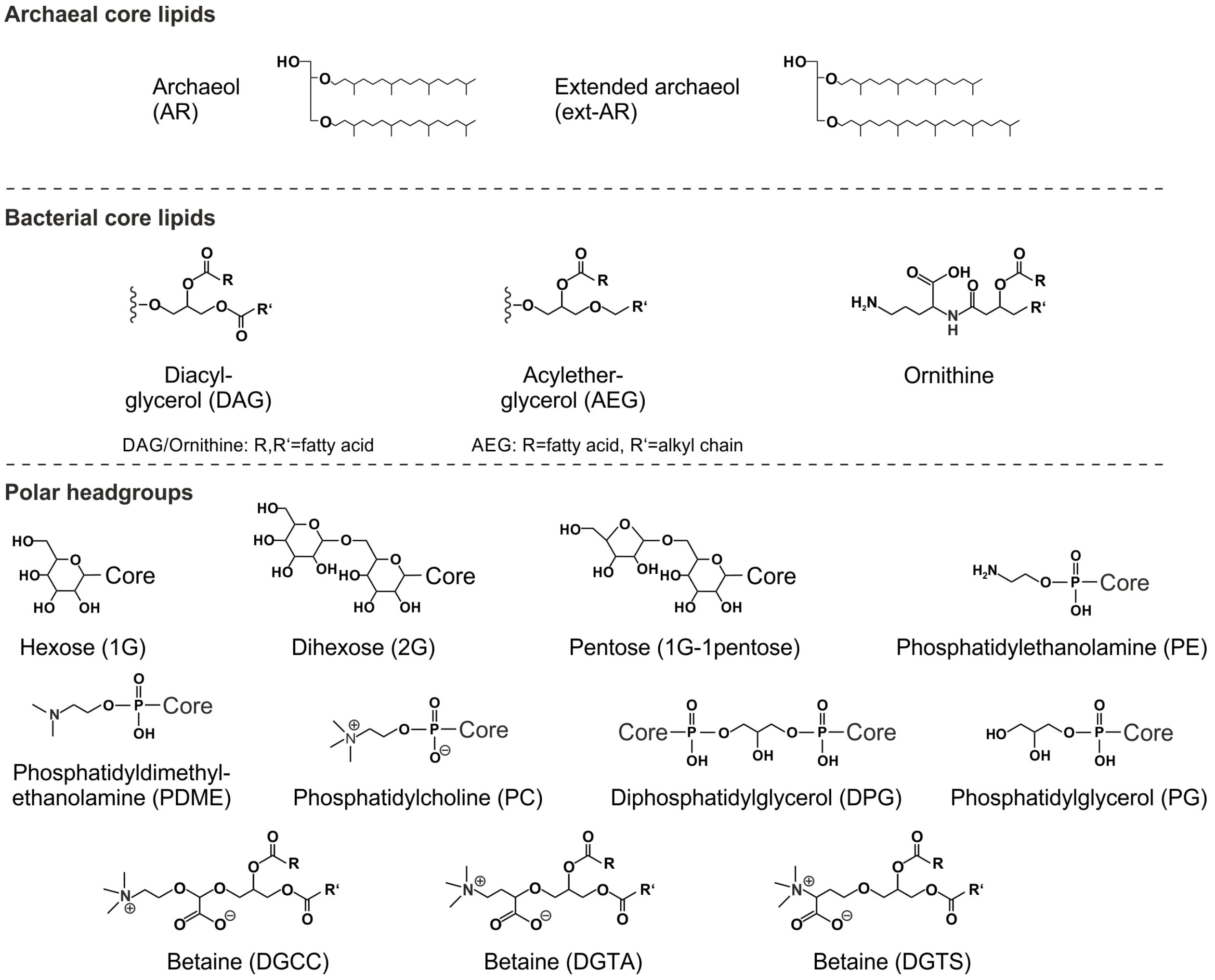

Fig. S3 | Elution pattern and fragmentation pattern of tentatively identified lyso-betaine lipids of types DGTS, DGTA, DGCC. a) Extracted ion chromatogram of lyso-DGTS-$C_{22:6}$ (exact mass *m/z* 546.3789 $[M+H]^+$), lyso-DGTA-$C_{22:6}$ (*m/z* 546.3789 $[M+H]^+$), and lyso-DGCC-$C_{22:6}$ (*m/z* 562.3738 $[M+H]^+$) in a total lipid extract of a recovery phase sample (sample 07, ~35% eruption cycle). b) Fragmentation of lyso-DGTS-$C_{22:6}$, lyso-DGTA-$C_{22:6}$, and lyso-DGCC-$C_{22:6}$.

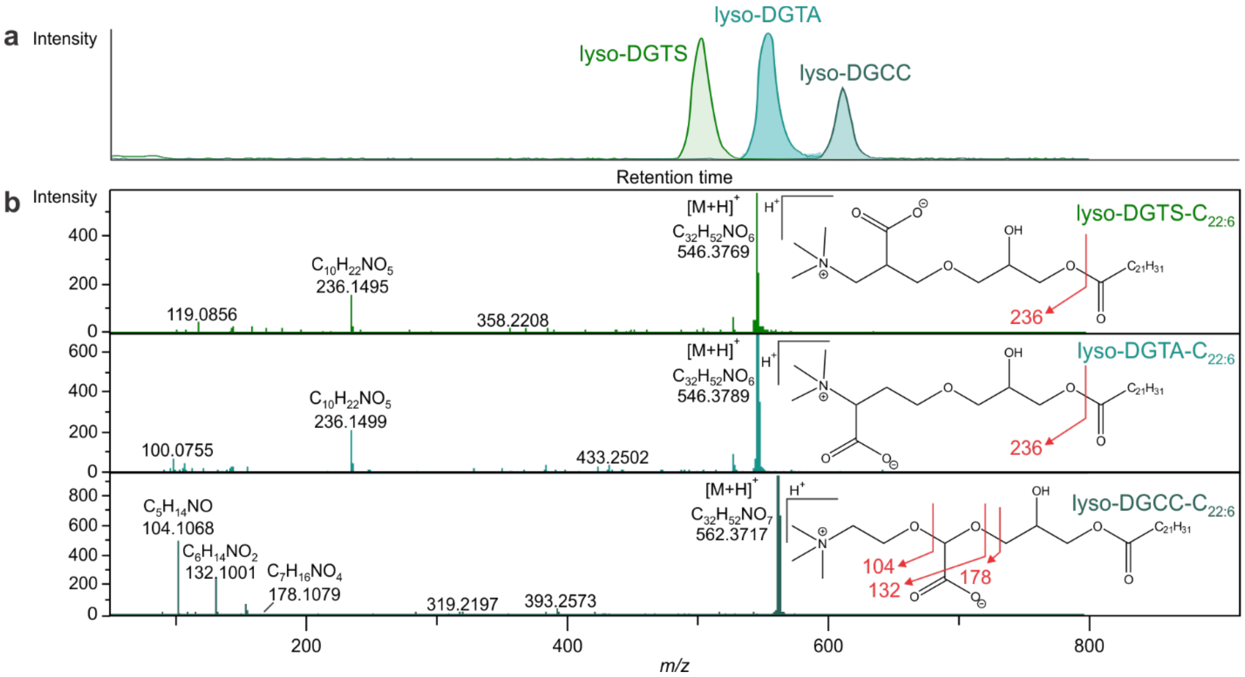

Fig. S4 | a) Extracted ion chromatogram of archaeal lipids detected in Crystal Geyser (sample 11, ~50% eruption cycle). b) Fragmentation and tentative structure of 1G-1pentose-AR (*m/z* 969.7577 $[M+H]^+$).

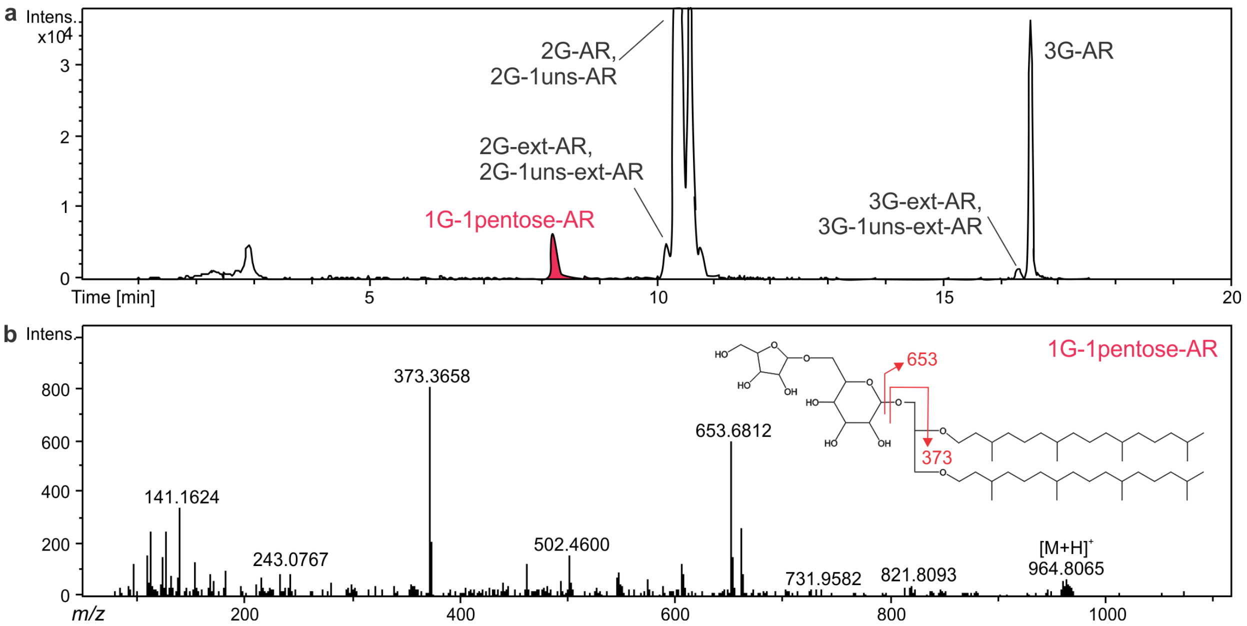

Fig. S5 | Percent relative abundance patterns of different rpS3-carrying scaffolds across the 27 metagenomic samples from Crystal Geyser (rpS3 sequences were clustered at 99% amino acid similarity, followed by stringent read-mapping for coverage calculation). Each sample is one line on the x-axis. Each color represents the relative abundance of one microbial rpS3 gene that was tracked over time. Coloring according to Fig. 1: Pink = recovery phase, green = minor eruptions, brown = major eruptions. Individual relative abundances are provided in Table S5.

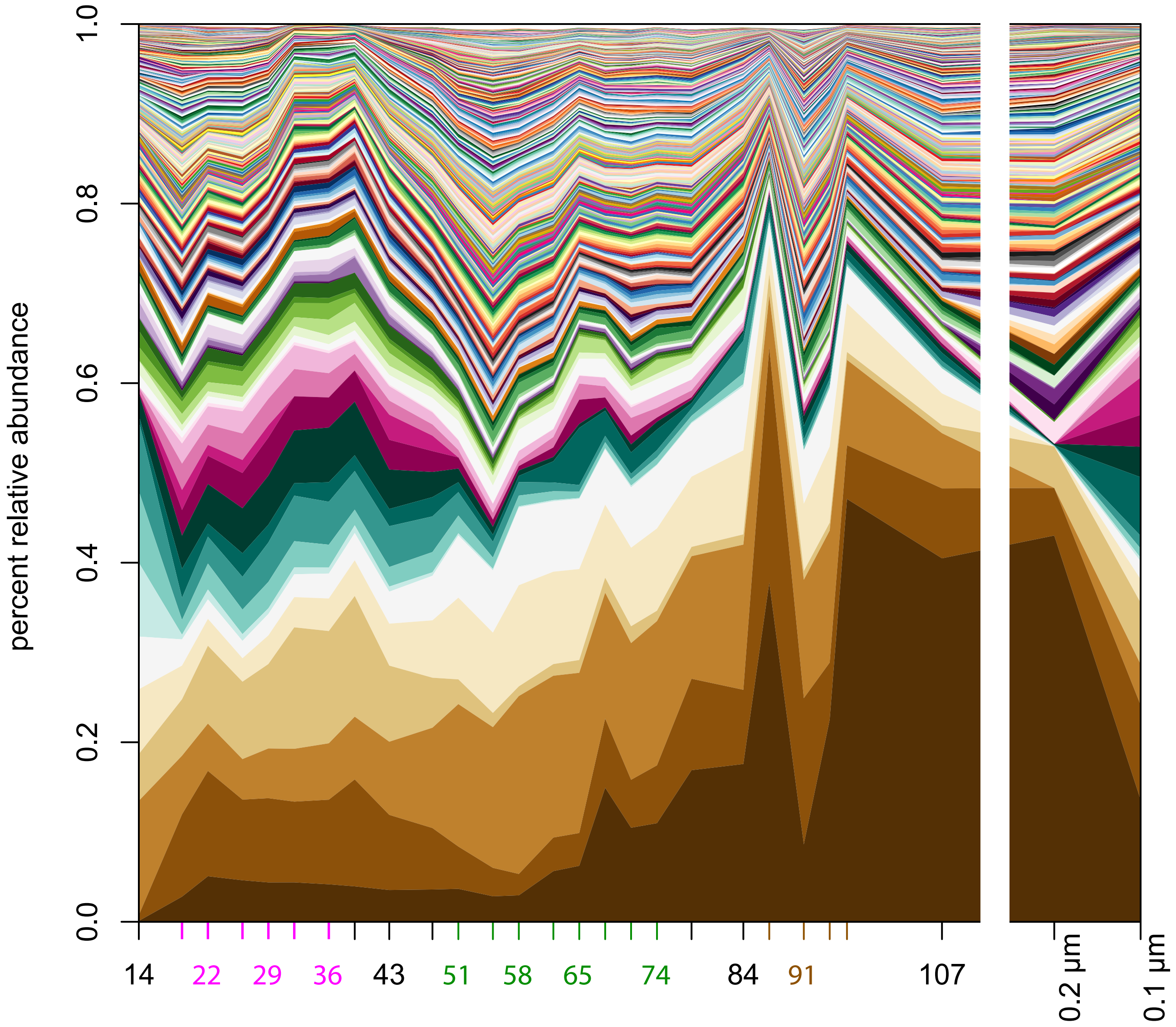

Fig. S6 | Water pressure (blue line) and $\delta^{13}C$ for DIC (red circles) sampled over the geyser cycle. The water pressure variations showing sourcing of fluids from the conduit (mixed), the deep aquifer, and the shallow aquifer from ref. [22].

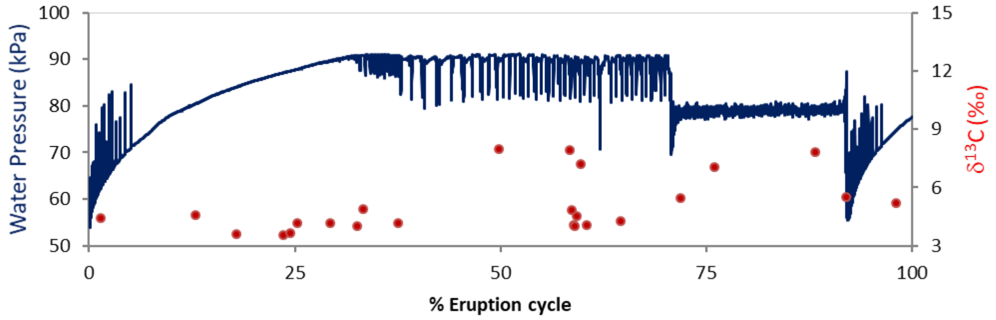

Fig. S7 | Percent relative abundance of scaffolds carrying predicted rpS3 sequences of Eukaryotes (identified via HMM profiles and searches, see Material and Methods). Samples are ordered by the eruption cycle and grouped as used for the correlation analysis.

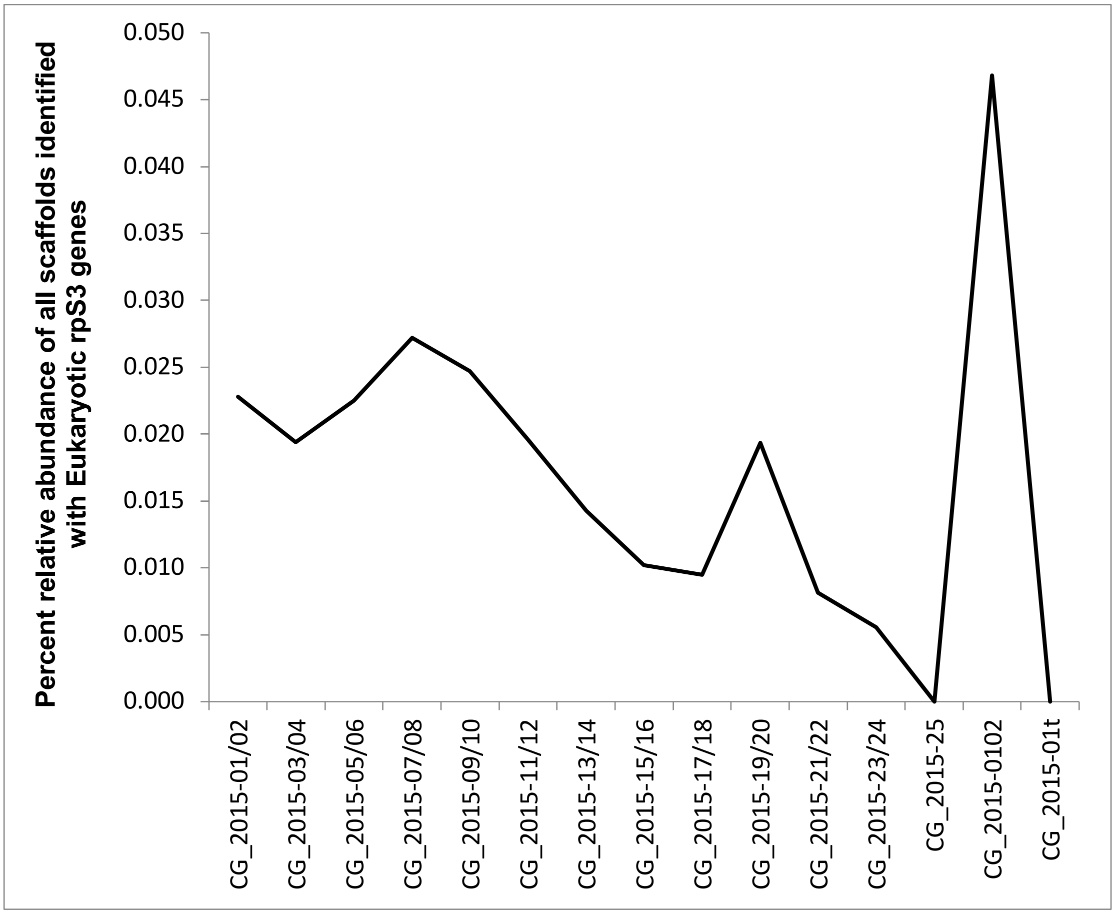

Fig. S8 | Diversity of 3-oxoacyl-[acyl-carrier protein] reductase beta subunit as a representative of the diversity of enzymes responsible for double-bond formation in fatty acid-based lipids. In total, 11,458 protein sequences of this enzyme were identified, which clustered into 1959 clusters at 90% similarity. For the displayed tree, four sequence clusters were excluded due to short alignment length. The 1954 clusters show a great diversity of different 3-oxoacyl-[acyl-carrier protein] reductases supporting the detected diversity of different fatty acid-based lipids with multiple double-bonds. Interestingly, many clusters with only one or few representatives were detected indicating an undersampling of the diversity of these dehydrogenases in the ecosystem.

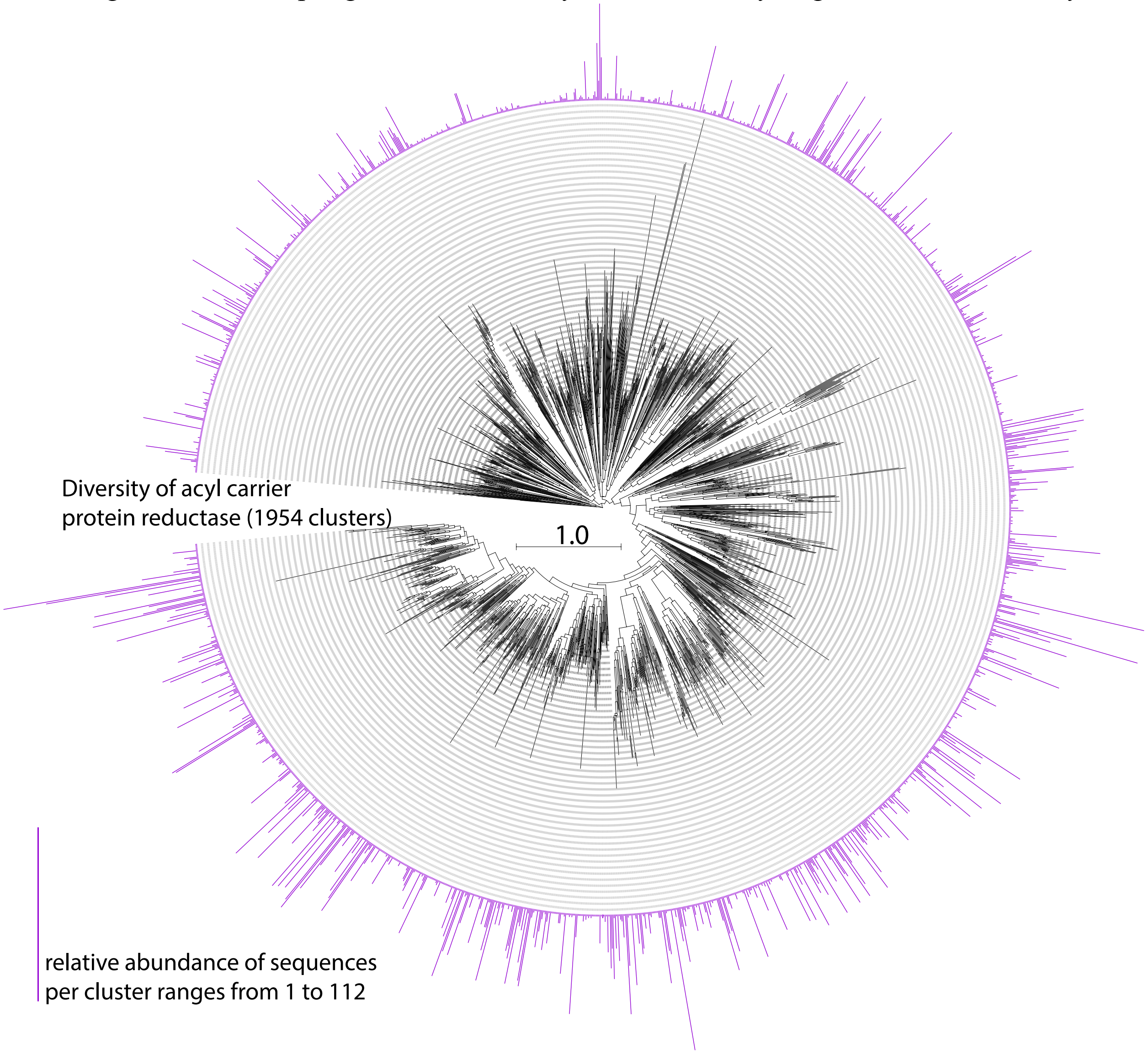

Fig. S9 | Correlations of relative abundance of *Altiarchaeum sp.* and archaeal IPLs in comparison with the standard *Huberiarchaeum crystalense* and its putative lipid. **A**. We detected three different rps3 genes of *Altiarchaeum sp.* (Table S5) in the samples highlighting the complexity of this species and the issues associated with its correlation analysis with IPLs. Moreover, the most abundant ether-based IPL (2G-AR), likely attributable to *Altiarchaeum sp.* as it is the most abundant species, also showed a presence (~1/10) in a post 0.2 µm filter but *Altiarchaeum sp.* did not. This indicates that *Altiarchaeum sp.* cells were lysing during the filtration process. **B**. Some individual lipids did correlate well with the total sum of the rpS3 sequences of *Altiarchaeum sp.* The example shown here is the typical lipid of *Altiarchaeum sp.* identified at another site [17]. **C**. *Huberiarchaeum crystalense* was used as a reference for a very stringent cutoff for q-values (FDR corrected p-values) due to the strain diversity and inconsistency of *Altiarchaeum sp.*

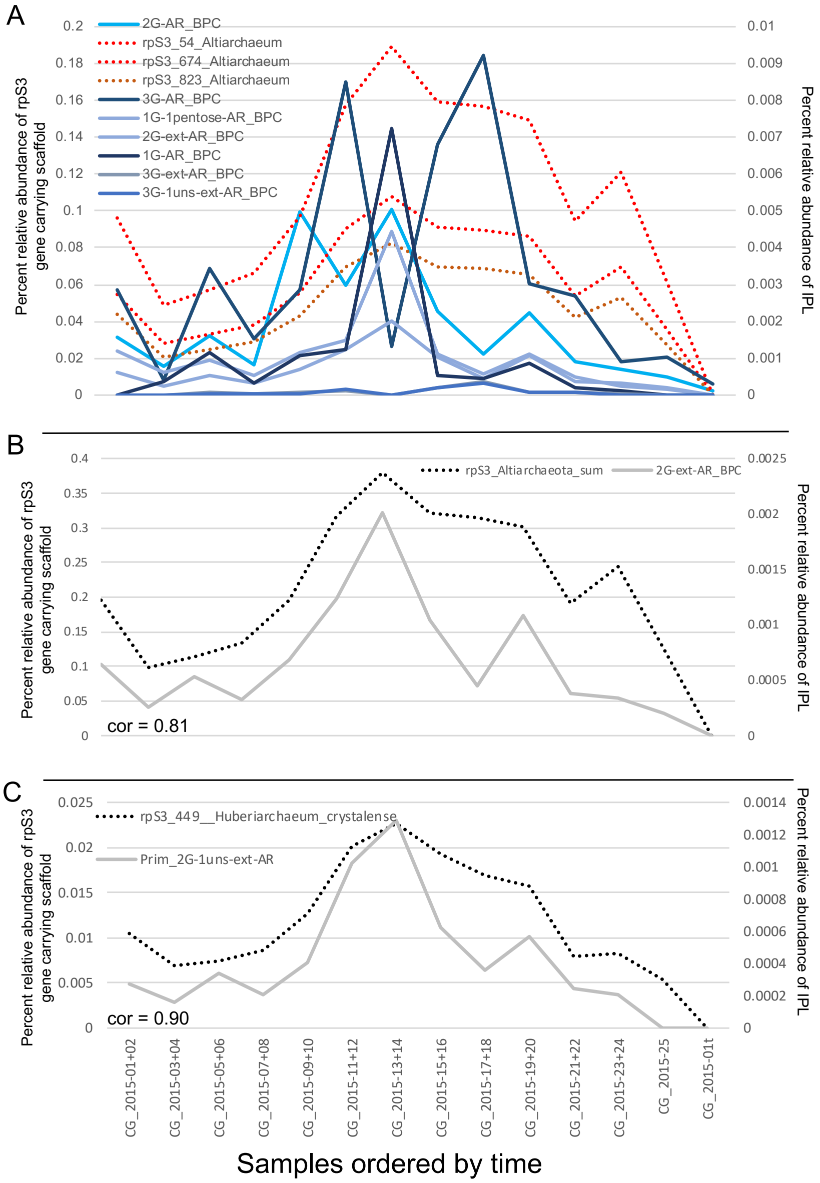

Fig. S10 | Rank abundance curve based on rpS3 sequences of the top50 organisms in the 0.1 µm filter used for infrared imaging. Taxonomic precisions are at domain and (super)phylum level. The majority of organisms were attributable to the CPR. X-axis displays the relative abundance based on read mapping.

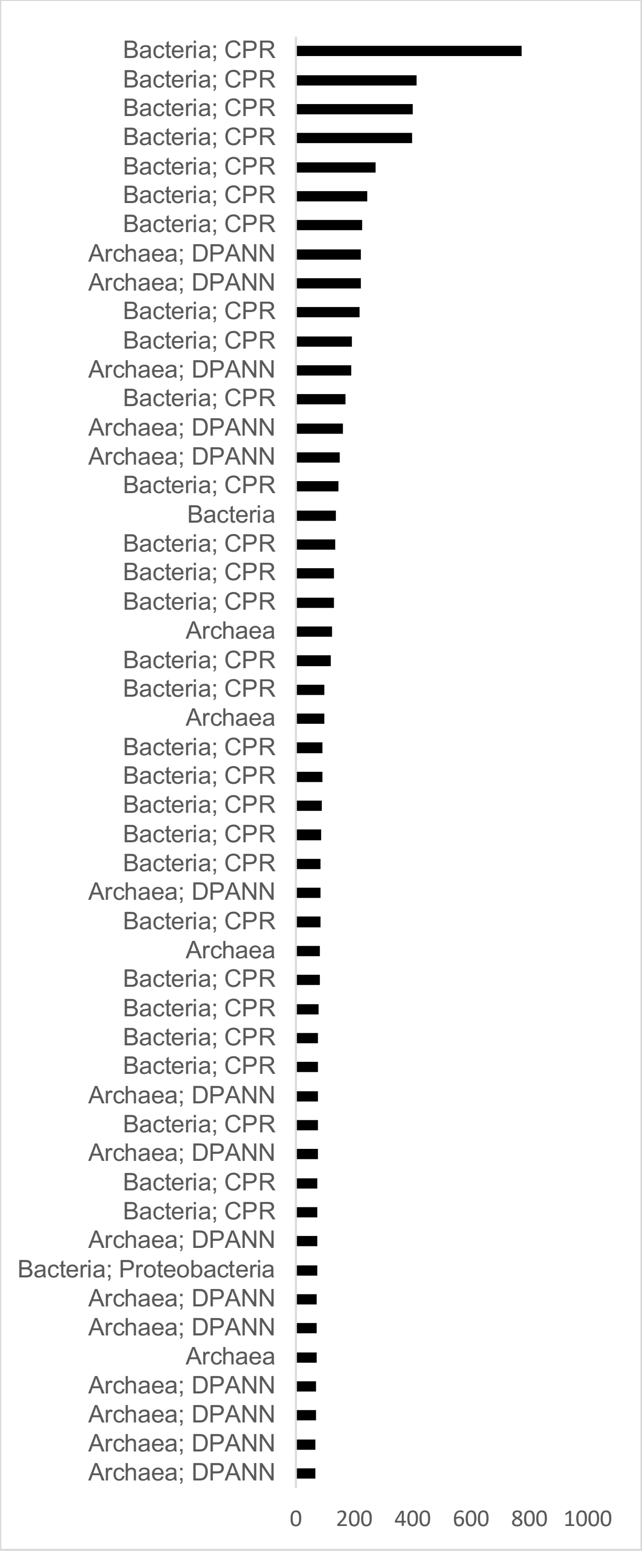

Fig. S11 | FTIR reference spectra of pure lipid and lysolipid standards.

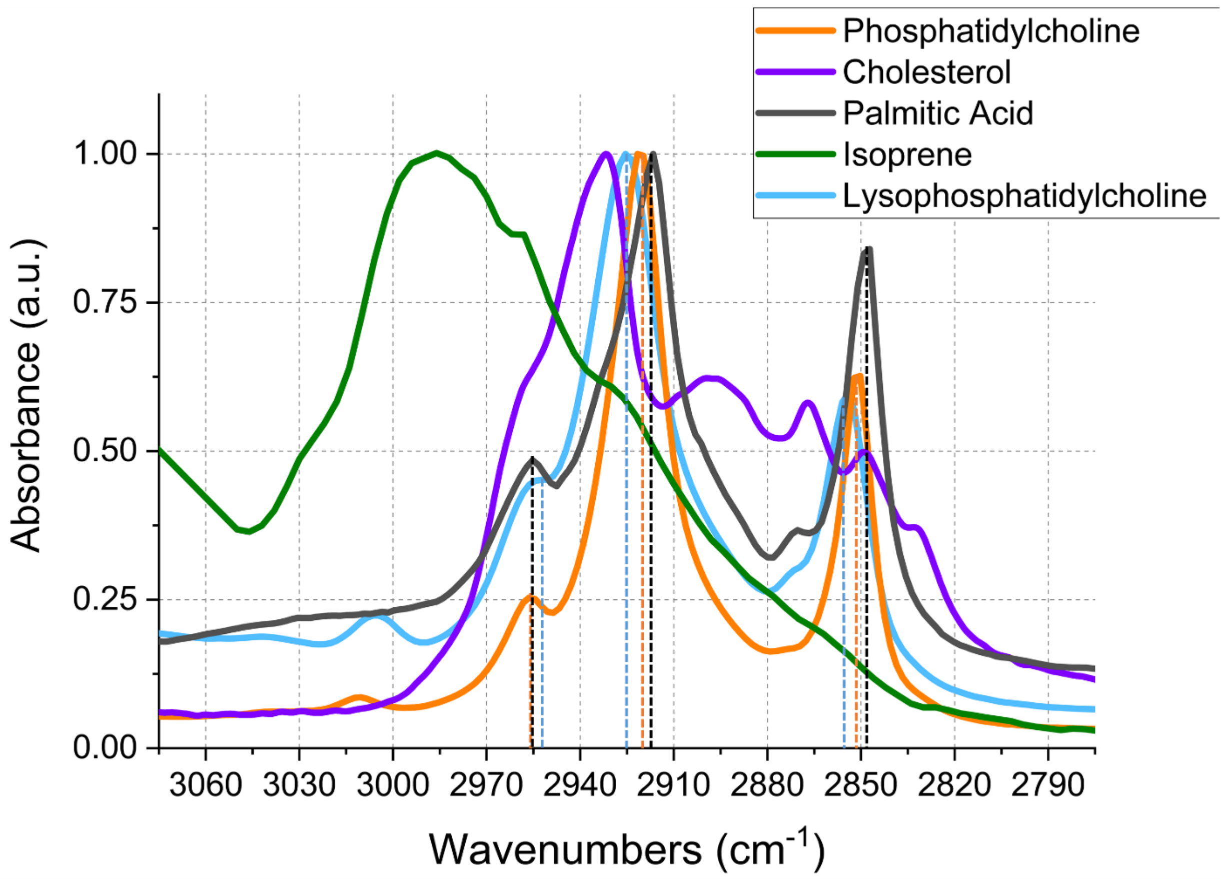

Fig. S12 | Loadings of the PCA over the whole 900-3700 cm^{-1} spectral range (a.u.: absorbance units) of FTIR measurements.

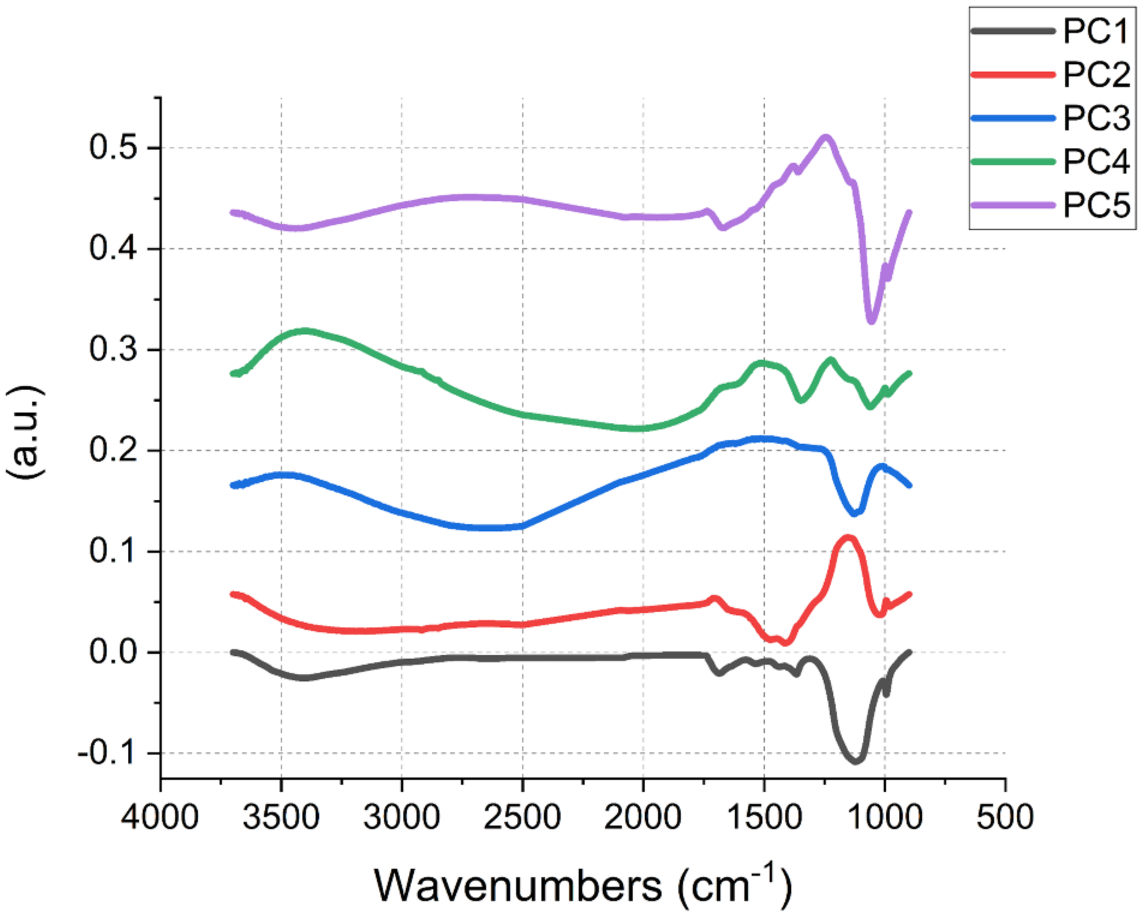

Table S4 | "Percent relative abundance and stable carbon isotopic composition of lipids across the cycle" is provided in a separate file.

Table S5 | "Percent relative abundance of rpS3 genes across the cycle" is provided in a separate file.

References

1. Sturt HF, Summons RE, Smith K, Elvert M, Hinrichs K-U. Intact polar membrane lipids in prokaryotes and sediments deciphered by high-performance liquid chromatography/electrospray ionization multistage mass spectrometry - new biomarkers for biogeochemistry and microbial ecology. *Rapid Communications in Mass Spectrometry* 2004; **18**: 617–628.
2. Wörmer L, Lipp JS, Schröder JM, Hinrichs K-U. Application of two new LC-ESI-MS methods for improved detection of intact polar lipids (IPLs) in environmental samples. *Organic Geochemistry* 2013; **59**: 10–21.
3. Becker KW, Elling FJ, Yoshinaga MY, Söllinger A, Urich T, Hinrichs K-U. Unusual Butane- and Pentanetriol-Based Tetraether Lipids in *Methanomassiliicoccus luminyensis*, a Representative of the Seventh Order of Methanogens. *Applied and Environmental Microbiology* 2016; **82**: 4505–4516.
4. Yoshinaga MY, Kellermann MY, Rossel PE, Schubotz F, Lipp JS, Hinrichs K-U. Systematic fragmentation patterns of archaeal intact polar lipids by high-performance liquid chromatography/electrospray ionization ion-trap mass spectrometry: Fragmentation patterns of archaeal intact polar lipids. *Rapid Communications in Mass Spectrometry* 2011; **25**: 3563–3574.
5. Elling FJ, Könneke M, Lipp JS, Becker KW, Gagen EJ, Hinrichs K-U. Effects of growth phase on the membrane lipid composition of the thaumarchaeon *Nitrosopumilus maritimus* and their implications for archaeal lipid distributions in the marine environment. *Geochimica et Cosmochimica Acta* 2014; **141**: 579–597.

6. Wörmer L, Lipp JS, Schröder JM, Hinrichs K-U. Application of two new LC–ESI–MS methods for improved detection of intact polar lipids (IPLs) in environmental samples. *Organic Geochemistry* 2013; **59**: 10–21.

7. Meador TB, Zhu C, Elling FJ, Könneke M, Hinrichs K-U. Identification of isoprenoid glycosidic glycerol dibiphytanol diethers and indications for their biosynthetic origin. *Organic Geochemistry* 2014; **69**: 70–75.

8. Summons RE, Franzmann PD, Nichols PD. Carbon isotopic fractionation associated with methylotrophic methanogenesis. *Organic Geochemistry* 1998; **28**: 465–475.

9. Elvert M, Boetius A, Knittel K, Jørgensen BB. Characterization of specific membrane fatty acids as chemotaxonomic markers for sulfate-reducing bacteria involved in anaerobic oxidation of methane. *Geomicrobiology Journal* 2003; **20**: 403–419.

10. Mook WG, Bommerson JC, Staverman WH. Carbon isotope fractionation between dissolved bicarbonate and gaseous carbon dioxide. *Earth and Planetary Science Letters* 1974; **22**: 169–176.

11. Berg IA, Kockelkorn D, Ramos-Vera WH, Say RF, Zarzycki J, Hügler M, et al. Autotrophic carbon fixation in archaea. *Nature Reviews Microbiology* 2010; **8**: 447–460.

12. Fuchs G. Alternative pathways of autotrophic CO_2 fixation. In: Schlegel HG, Bowien B (eds). *Biology of Autotrophic Bacteria*. 1989. Springer, Berlin, pp 365–382.

13. Quandt L, Gottschalk G, Ziegler H, Stichler W. Isotope discrimination by photosynthetic bacteria. *FEMS Microbiology Letters* 1977; **1**: 125–128.

14. McNevin DB, Badger MR, Whitney SM, Von Caemmerer S, Tcherkez GGB, Farquhar GD. Differences in carbon isotope discrimination of three variants of D-ribulose-1,5-bisphosphate

carboxylase/oxygenase reflect differences in their catalytic mechanisms. *Journal of Biological Chemistry* 2007; **282**: 36068–36076.

15. Preuß A, Schauder R, Fuchs G, Stichler W. Carbon isotope fractionation by autotrophic bacteria with three different CO_2 fixation pathways. *Zeitschrift für Naturforschung* 1989; **44**: 397–402.
16. Sirevåg R, Buchanan BB, Berry JA, Troughton JH. Mechanisms of CO_2 Fixation in Bacterial Photosynthesis Studied by the Carbon Isotope Fractionation Technique. *Arch Microbiol* 1977; **112**: 35–38.
17. Probst AJ, Weinmaier T, Raymann K, Perras A, Emerson JB, Rattei T, et al. Biology of a widespread uncultivated archaeon that contributes to carbon fixation in the subsurface. *Nature Communications* 2014; **5**: 5497.
18. Beleites C, Sergo V. hyperSpec: a package to handle hyperspectral data sets in R. 2018.
19. signal developers. signal: Signal processing. 2014.
20. Liland K, Mevik B. baseline: Baseline correction of spectra.
21. Lalonde K, Middlestead P, Gélinas Y. Automation of $^{13}C/^{12}C$ ratio measurement for freshwater and seawater DOC using high temperature combustion. *Limnology and Oceanography: Methods* 2014; **12**: 816–829.
22. Probst AJ, Ladd B, Jarett JK, Geller-McGrath DE, Sieber CMK, Emerson JB, et al. Differential depth distribution of microbial function and putative symbionts through sediment-hosted aquifers in the deep terrestrial subsurface. *Nature Microbiology* 2018; 1.
23. West PT, Probst AJ, Grigoriev IV, Thomas BC, Banfield JF. Genome-reconstruction for eukaryotes from complex natural microbial communities. *Genome Res* 2018; gr.228429.117.

24. Probst AJ, Castelle CJ, Singh A, Brown CT, Anantharaman K, Sharon I, et al. Genomic resolution of a cold subsurface aquifer community provides metabolic insights for novel microbes adapted to high CO_2 concentrations. *Environmental Microbiology* 2017; **19**: 459–474.

25. Hyatt D, Chen G-L, LoCascio PF, Land ML, Larimer FW, Hauser LJ. Prodigal: prokaryotic gene recognition and translation initiation site identification. *BMC Bioinformatics* 2010; **11**: 119.

26. Finn RD, Clements J, Eddy SR. HMMER web server: interactive sequence similarity searching. *Nucleic Acids Res* 2011; **39**: W29–W37.

27. Suetake H, Kotera M. KPHMMER: Hidden Markov Model generator for detecting KEGG PATHWAY-specific genes. *bioRxiv* 2019; 636290.

28. Li W, Godzik A. Cd-hit: a fast program for clustering and comparing large sets of protein or nucleotide sequences. *Bioinformatics* 2006; **22**: 1658–1659.

29. Edgar RC. MUSCLE: multiple sequence alignment with high accuracy and high throughput. *Nucleic Acids Res* 2004; **32**: 1792–1797.

30. Price MN, Dehal PS, Arkin AP. FastTree 2 – Approximately Maximum-Likelihood Trees for Large Alignments. *PLOS ONE* 2010; **5**: e9490.

31. Letunic I, Bork P. Interactive Tree Of Life (iTOL) v4: recent updates and new developments. *Nucleic Acids Res* 2019; **1**.
